# Supplementary material for: Applying gradient tree boosting to QTL mapping with Shapley additive : explanations
Source: Breed Sci. 2025 Oct 29;75(5):378–91. doi: 10.1270/jsbbs.24083 (PMC13129572; doi:10.1270/jsbbs.24083)
Supplement: Supplementary file 2 — Supplemental Table [file 75_378_s2.pdf]

doi: 10.1270/jsbbs.24083

Supplemental Table 1 QTLs and QTL interactions detected with multiple interval mapping in the real rice data

Environment	Main effects				Interact with			
	Chr	Position (cM)	Major gene	Size	Chr	Position (cM)	Major gene	Size
Ishikawa2008	3	95.33	*Hd6*	0.6362				
	5	68.73		2.2831				
	6	30.57	*Hd1*	-7.9966				
	7	91.5	*Hd2*	-1.6585				
	8	18.73	*DTH8*	8.0201				
	11	3.85		1.1193				
Tsukuba2007	1	35.44		1.4103				
	3	94.45	*Hd6*	0.6988				
	5	68.73		1.8493				
	6	30.57	*Hd1*	-6.0632				
	7	91.5	*Hd2*	-1.1049				
	8	22.54	*DTH8*	5.7596				
Tsukuba2008E	3	96.19	*Hd6*	0.1404				
	5	68.73		1.8944				
	6	30.57	*Hd1*	-8.7395				
	7	91.5	*Hd2*	-2.6196				
	8	22.54	*DTH8*	7.8378				
	11	3.85		0.6623				
Tsukuba2008L	3	122.63	*Hd6*	3.6521	7	91.5	*Hd2*	-2.753
	6	30.57	*Hd1*	-3.4591				
	7	25.8		1.3684				
	7	91.5	*Hd2*	0.8667				
	8	17.83	*DTH8*	5.2241				
	11	3.85		0.1474				
Tsukuba2009	3	98.67	*Hd6*	0.5534				
	5	68.73		2.1994				
	6	30.57	*Hd1*	-8.18				
	7	91.5	*Hd2*	-2.1135				
	8	18.73	*DTH8*	7.4722				
	11	1.97		1.0456				

(Table S1 continued)

Environment	Main effects				Interact with			
	Chr	Position (cM)	Major gene	Size	Chr	Position (cM)	Major gene	Size
Fukuoka2008	1	35.43		1.2397				
	3	124.69	*Hd6*	2.9079	6	30.57	*Hd1*	-3.026
					7	83.59	*Hd2*	-2.977
	6	30.57	*Hd1*	-6.5639				
	7	25.89		1.616				
	7	83.59	*Hd2*	1.584	8	18.72	*DTH8*	2.435
	8	18.72	*DTH8*	6.3669				
Ishigaki2008	1	35.44		-5.3063	11	3.85		-6.482
	3	117.23	*Hd6*	2.1597	7	91.5	*Hd2*	-1.565
	6	13.02		-0.3499				
	7	91.5	*Hd2*	5.0085	8	13.07	*DTH8*	2.529
	8	13.07	*DTH8*	4.6862				
	11	3.85		-5.8289				
ThaiNguyn2008	1	4.77		-2.4888				
	3	117.23	*Hd6*	1.8787				
	6	12.03		0.743				
	7	91.5	*Hd2*	4.4247				
	8	15.03	*DTH8*	3.4037				
	11	0.01		-1.1966				
HaNoi2008	3	96.19	*Hd6*	4.9436	8	22.54	*DTH8*	5.721
	6	30.57	*Hd1*	2.474				
	7	86.39	*Hd2*	2.4728				
	8	22.54	*DTH8*	2.2514	10	46.71		-4.793
	10	46.71		-4.8473				
	11	3.85		0.3141				
